# Evaluation of Antidiabetic Activity of the Leaf Latex of *Aloe pulcherrima* Gilbert and Sebsebe (Aloaceae)

**DOI:** 10.1155/2020/8899743

**Published:** 2020-10-03

**Authors:** Gedefaw Getnet Amare, Birhanu Geta Meharie, Yaschilal Muche Belayneh

**Affiliations:** Department of Pharmacy, College of Medicine and Health Sciences, Wollo University, Dessie, Ethiopia

## Abstract

The leaf latex of *Aloe pulcherrima* has been used as remedy for diabetes mellitus. This was carried out to determine *in vitro* and *in vivo* antidiabetic activities of the leaf latex of *Aloe pulcherrim*a. *Methods*. Sucrase and maltase inhibitory activity of the leaf latex of *A. pulcherrima* was determined in glucose oxidase assay, and *α*-amylase inhibitory activity was determined in dinitrosalicylic acid assay. Normoglycemic, glucose-loaded, and streptozotocin-induced diabetic mice were treated orally to determine blood glucose lowering activity of the latex. Effect of the latex on serum lipid level and body weight was measured in streptozotocin-induced diabetic mice. Additionally, DPPH assay was used to determine free radical scavenging capacity of the latex. *Results*. Antioxidant activity of the latex was concentration dependent; the strongest inhibition was measured at 800 *μ*g/ml (80.57%). The leaf latex of *A. pulcherrima* inhibited sucrase (IC_50_ = 2.92 *μ*g/ml), maltase (IC_50_ = 11.81 *μ*g/ml) and *α*-amylase (IC_50_ = 14.92 *μ*g/ml) enzymes. All doses of the leaf latex induced hypoglycemic effect after 4 h in normal mice, and low dose of the latex did not show significant effect after 6 h. Glucose reduction of the leaf latex of *A. pulcherrima* was significant (*p* < 0.05) in oral glucose-loaded mice compared to the vehicle control. Blood glucose level of diabetic mice was significantly (*p* < 0.05) reduced on week one and weak two in a streptozotocin-induced diabetic mouse model. Glucose reduction increased with increasing the doses of the leaf latex of *A. pulcherrima* on week one (*p* < 0.05 (200 mg/kg), *p* < 0.01 (400 mg/kg), and *p* < 0.001 (600 mg/kg)). Administration of the leaf latex of *A. pulcherrima* for two weeks significantly (*p* < 0.05) improved diabetic dyslipidemia and body weight of diabetic mice. *Conclusion*. The study confirmed that the leaf latex of the plant showed a significant antidiabetic activity justifying the traditional uses of the plant.

## 1. Background

Herbal therapies are proven safe and effective for healing diseases and have been the potential source for the development of new drugs [1, 2]. A majority of people in the world rely on herbal therapy [1, 3]. Phytotherapy is the antecedent of modern drugs, and one-third of the top-selling drugs in the world are plant origins [4, 5]. More than 800 plants have been used as remedies for diabetes healing, and the most successful story in drug discovery is isolation of guanidines (analogue of metformin) from *Galega officinalis* [2, 6]. A variety of phytochemicals such as flavonoids, phenols, triterpenoids, and alkaloids have shown prominent antidiabetic activity [2, 7, 8]. The leaf of *Capparis spinosa* L. and *Juglans regia* L. contained phenols and flavonoids (rutin) that showed prominent antidiabetic and antidyslipidemic activity [9, 10]. Rutin showed strong free radical scavenging and cholinesterase inhibitory activity and reverse kidney and liver injuries [10, 11]. The leaf gel of *Aloe vera* offers promising results for the development of novel antidiabetic drugs. The leaf gel of this plant has been under clinical trial in prediabetic and type 2 diabetic patients [12, 13]. Combination of deleterious effects, limited efficacy, and contraindication of current antidiabetic drugs initiate noble antidiabetic drug development to minimize DM progression and deleterious effects [14–16].

**Figure 1:**
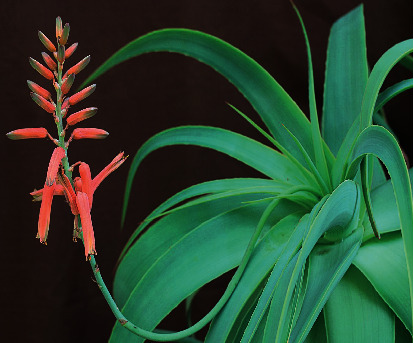
Picture of *Aloe pulcherrima*.

*Aloe pulcherrima*, which belongs to Aloaceae plant family, is an Ethiopian endemic plant. The plant shows U-shaped peduncle in its flowers (Figure 1) [17]. Many compounds including chrysophanol, aloesaponarin, nataloin, and 7-hydroxyaloin were isolated from the plant and showed antibacterial, antifungal, and antimalarial effects [18, 19]. The leaf latex of *Aloe pulcherrima* was found to be safe up to 5000 mg/kg in mice [18]. The leaf latex of the plant has been used in folk medicine to treat diabetes mellitus without scientific studies [20, 21]. Therefore, the current study was conducted to investigate antidiabetic activity of the leaf latex of plant.

## 2. Methods

### 2.1. Chemicals

Ascorbic acid (99%, Japan), dinitrosalicylic acid, phenol, Na_2_SO_3_, sodium hydroxide, potassium sodium tartrate, phosphate, sucrose, maltose, intestinal acetone powder, DPPH (97%, Tokyo, Japan), citric acid monohydrate, and trisodium citrate dihydrate were used during the study.

### 2.2. Preparation of the Leaf Latex of *Aloe pulcherrima*

The leaf of *Aloe pulcherrima* Gilbert and Sebsebe was collected from Fiche (located in Oromia Region, Central Ethiopia) in December, 2019. The plant was identified by Professor Sebsebe Demisew (botanist), and the specimen was deposited in the National Herbarium of Addis Ababa University (AAU) with the voucher number of SD009/19. The leaf of *Aloe pulcherrima* was cut transversely close to the stem, and then, the leaf was inclined towards the collecting plate to obtain yellowish exudate. The latex was dried under shade at room temperature with optimal ventilation. The dried latex was crushed into powder by mortar and pestle and stored in vial until used for the experiment.

### 2.3. Experimental Mice

Male Swiss albino mice (weighing 25–35 grams and 2-3 months of age) were purchased from EPHI and used in the study. Mice were maintained at 12 h light/dark cycle and were allowed free to standard pellet diet and water *ad libitum*.

### 2.4. Phytochemical Analysis of the Leaf Latex of *Aloe pulcherrima*

Qualitative preliminary phytochemical screening tests were carried out for the dried latex of *Aloe pulcherrima* as per the standard methods to analyze the presence or absence of secondary metabolites [22, 23].

### 2.5. Antioxidant Activity of the Leaf Latex of *Aloe pulcherrima*

Antioxidant capacity of the latex was evaluated in DPPH assay as described in the literature with some modifications [24]. Three milliliters of 0.1 mM (4 mg/100 ml solution) was mixed with one milliliter of methanolic solution of different concentrations (25–800 *μ*g/ml) of the latex. Ascorbic acid with the same concentration was served as the positive control, and 4 ml solution of the free radical (DPPH) without the latex was used as the vehicle control. After 30-minute incubation, the absorbance of test solutions and the control were read at 517 nm via a UV spectrophotometer. Percentage was calculated by the following formula:

(1) $$\begin{array}{c} \%\text{ of free radical inhibition}=\frac{\left(A_{0}-A_{1}\right)}{A_{0}}\times 100, \end{array}$$

where *A*_0_ is absorbance of the control and *A*_1_ is absorbance of the extract/standard.

### 2.6. Enzyme Inhibitory Measurement

The *α*-amylase inhibitory assay of the leaf latex of *A. pulcherrima* was determined as mentioned in the literature studies with few modifications [24–26]. Ten *μ*l latex solution (15.6–500 *μ*g/ml) was incubated in 75 *μ*l porcine pancreatic *α*-amylase (3 units/ml) in 165 *μ*l phosphate buffers (pH 6.9, 0.1 M) at 37°C for 15 minutes. A volume of 100 *μ*l starch solution (1 g/L) in buffer was added to the reaction mixture and incubated at 37°C for 20 min. The reaction was ceased by adding 250 ml dinitrosalicylic acid (1%) and boiled in water bath for ten minute at 100°C. Then, 250 *μ*l potassium sodium tartrate (40%) was added into the mixtures. Acarbose was served as a positive control, and IC_50_ was determined. The mixtures were kept on cold water bath at room temperature, and the absorbance was read at 540 nm by using by a UV spectrometer:

(2) $$\begin{array}{c} \%\text{ of free radical inhibition}=\frac{\left(A-B\right)}{A}\times 100, \end{array}$$

where *A* is the absorbance of the control and *B* is the absorbance of the latex/standard.

The sucrase and maltase inhibitory assays of the leaf latex were determined by the glucose oxidase method [26]. Acetone solution (100 mg in 3 ml normal saline) was centrifuged at 12,000 rpm for thirty minutes. A volume of 30 *μ*l sucrase and 10 *μ*l maltase solutions was incubated in 40 *μ*l sucrose (400 mM) and 30 *μ*l maltose (86 mM) solutions, respectively. Ten *μ*l latex solution (15.6–500 *μ*g/ml) and then 0.1 M phosphate buffer (pH = 6.9) were added to maintain the 100 *μ*l final volume and incubated for 1 hr at 37°C. Then, the mixtures were suspended in boiling water for ten minutes to cease the reaction. The absorbance of glucose which was released from the reaction was read at 450 nm by the glucose oxidase method. Sucrase and maltase inhibitory activities were represented as % inhibition:

(3) $$\begin{array}{c} \%\text{ inhibition}=\frac{\left(A-B\right)}{A}\times 100, \end{array}$$

where *A* is the absorbance of the control and *B* is the absorbance of the samples.

### 2.7. Grouping and Dosing of Animals

Male mice were used in the normoglycemic, oral glucose tolerance test (OGTT) and streptozotocin-induced diabetic model since female mice have low survival, insensitive to STZ and insulin [27–29]. In normoglycemic and OGTT, mice were randomly divided into five groups (*n* = 6). Groups 1 and 2 (negative and positive control) were treated with 10 mg/kg vehicle (2% tween 80 in distilled water) and 5 mg/kg glibenclamide (Julphar), respectively; groups 3, 4, and 5 were given 200,400, and 600 mg/kg of the leaf latex. In the STZ-induced diabetic model, mice were randomly divided into six groups (5 diabetic groups and 1 nondiabetic group, *n* = 6) [30]. Groups 1 and 2 (normal and diabetic control) were given the vehicle, and group 3 was treated with standard drug. Groups 4, 5, and 6 (diabetic test groups) were treated with 200, 400, and 600 mg/kg of the leaf latex.

### 2.8. Induction of Experimental Diabetes

Experimental diabetes was induced by intraperitoneal injection of streptozotocin at the dose of 150 mg/kg on 16 h fasted mice [30, 31]. After thirty-minute STZ injection, mice were allowed free to food and water. After six hours, mice were allowed *ad libitum* glucose solution (5%) for one day to prevent hypoglycemic shock and death. After three days of streptozotocin administration, mice with fasting BGL >200 mg/dl were candidate for the experiment [27, 31–33].

### 2.9. Evaluation of the Effect of the Leaf Latex in Normoglycemic Model

Sixteen-hour fasted normal mice were treated orally with vehicle, standard drug, and different doses of the leaf latex according to their respective groups as mentioned above. Blood was measured from the tail vein of the mice by using a glucometer with strip to determine the glucose level before treatment and at 1, 2, 4, and 6 h after treatment [27, 34].

### 2.10. Evaluation of the Effect of the Leaf Latex on Oral Glucose Tolerance Test (OGTT)

The oral glucose tolerance test was done in sixteen-hour fasted mice to evaluate antihyperglycemic potential of the leaf latex of *A. pulcherrima* [30, 35, 36]. Fasted mice were treated with the vehicle, glibenclamide, and the three different doses of the latex according to their groups. After thirty minutes, oral glucose solution (40%) at the dose of 2 g/kg was given to the mice. Then, blood was withdrawn from the tail vein of each mouse to determine the glucose level at 0 h (before glucose administration) and at 0.5, 1, and 2 h after glucose administration [27, 30, 31].

### 2.11. Evaluation of the Effects of the Leaf Latex on Blood Glucose, Body Weight, and Plasma Lipid Level of STZ-Induced Diabetic Mice

After 3 days of streptozotocin administration, normal and diabetic mice were randomly assigned to different groups and then BGL and body weight of the mice were measured. Then, mice were treated with the vehicle, standard drug, and the latex daily for two weeks. On weeks one and two, BGL and body weight of 16-hour fasted mice were recorded [31, 37]. On day 15, overnight fasted mice were sacrificed using high-dose pentobarbitone (150 mg/kg, i.p.) and then blood samples were collected from each mouse in a sterile tube. Blood samples were allowed to coagulate for two hours at room temperature, then centrifuged at 2000 rpm for ten minutes. Then, serum samples were prepared by decanting the supernatant into test tubes to determine the level of TC, TG, and HDL-C.

### 2.12. Experimental Animal Sacrification (Euthanasia)

Diabetic mice were sacrificed at the end of the experiment by using a high-dose anesthetic agent. Each mouse was anesthetized by pentobarbitone at the dose of 150 mg/kg intraperitoneally [38].

### 2.13. Statistical Analysis

The results of the study were expressed as mean ± standard error of the mean. Statistical analysis of the data was carried out with one-way analysis of variance followed by the Tukey post hoc multiple comparison test. Significant differences were set at *p* values lower than 0.05.

## 3. Results

### 3.1. Phytochemical Analysis of the Leaf Latex of *Aloe pulcherrima*

In this study, preliminary phytochemical analysis indicated the presence of the following secondary metabolites in the leaf latex of the plant (Table 1).

### 3.2. Antioxidant Activity of the Leaf Latex of *Aloe pulcherrima*

**Figure 2:**
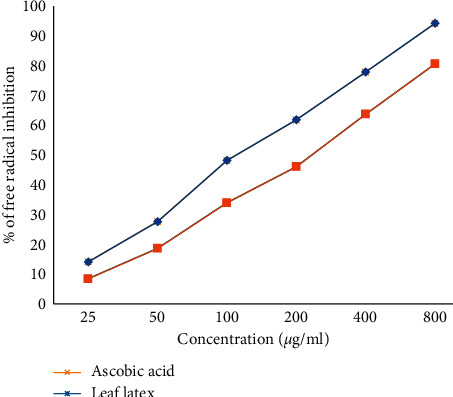
Percentage of free radical inhibition activity of the leaf latex of *A. pulcherrima*.

The leaf latex of *A. pulcherrima* showed concentration-dependent antioxidant activity in DPPH assay (Figure 2). The finding showed that the color of tested solution was changed from violet to slight yellow depending on the concentration. The latex was endowed with antioxidant activity, and 50% inhibitory concentration (IC_50_) was found to be 9.36 *μ*g/ml (*p* < 0.001).

### 3.3. The Inhibitory Effect of the Leaf Latex of *Aloe pulcherrima* on Sucrase, Maltase, and *α*-Amylase Enzymes

**Figure 3:**
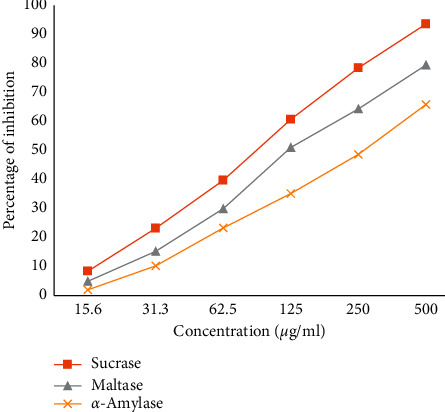
The effect of the leaf latex of *Aloe pulcherrima* on sucrase, maltase, and *α*-amylase enzymes.

Enzyme inhibition assay showed that the latex possessed an inhibitory effect on sucrase, maltase and *α*-amylase enzymes (Figure 3). Even though all enzymes were inhibited by the latex, greater inhibition was shown on sucrase (IC_50_ = 2.92 *μ*g/ml) than maltase (IC_50_ = 11.81 *μ*g/ml) and *α*-amylase (IC_50_ = 14.92 *μ*g/ml) enzymes. The finding of the study showed that sucrase, maltase, and *α*-amylase inhibition capacity of the latex was concentration dependent. Percentage inhibition of the latex was significant (*p* < 0.001) compared to vehicle control.

### 3.4. The Effect of the Leaf Latex of *Aloe pulcherrima* on Glucose Level in Normoglycemic Mice

Four hours after administration of the latex, all doses showed significant (*p* < 0.05) hypoglycemic effect compared to vehicle control (Table 2). After 6 h, hypoglycemic effect of 400 and 600 mg/kg dose was significant (*p* < 0.01) in normal mice but 200 mg/kg of the latex did not show hypoglycemic effect at 6^th^ h after treatment. In addition, glibenclamide (5 mg/kg) produced significant effect (hypoglycemia) at 2 (*p* < 0.01), 4 (*p* < 0.01), and 6 h (*p* < 0.001) compared to the vehicle control.

### 3.5. Effect of the Leaf Latex of *A. pulcherrima* on Glucose Tolerance Test in Mice

Mean BGL of all groups was not significantly different from each other at zero and thirty minutes after glucose feeding (Table 3). Peak blood glucose level was measured after thirty minutes. Blood glucose reduction was significant (*p* < 0.001) at 1 and 2 hours with 400 and 600 mg/kg of the leaf latex compared to the vehicle. Blood glucose reduction at 200 mg/kg dose of the latex was delayed and found to be significant (*p* < 0.01) after 2 hours.

### 3.6. Effect of the Latex of *A. pulcherrima* on Blood Glucose Level of STZ-Induced Diabetic Mice

Dose-dependent blood glucose reduction was measured in the mice treated with all doses of the latex on week one and week two compared to diabetic control. Glucose level of the mice treated with the latex at 200 mg/kg was significantly (*p* < 0.05 on week 1; *p* < 0.01 on week 2) changed compared to diabetic control, but glucose reduction was significantly (*p* < 0.05) lower than 5 mg/kg glibenclamide (Table 4). Blood glucose reduction with 400 mg/kg (*p* < 0.01 on week 1; *p* < 0.001 on week 2) and 600 mg/kg (*p* < 0.001 on both weeks) of the latex was significant compared to the vehicle control.

### 3.7. Effect of the Leaf Latex of *Aloe pulcherrima* on Body Weight of STZ-Induced Diabetic Mice

Significant body weight loss was measured in diabetic control compared to the mice treated with all doses of the latex and glibenclamide. The three doses of the latex significantly (*p* < 0.01) improved body weight of the mice on weeks one and two compared to vehicle control (Table 5). There was no significant body weight variation within latex- and glibenclamide-treated groups.

### 3.8. Effect of the Leaf Latex of *Aloe pulcherrima* on Serum Lipid Level in Diabetic Mice

Serum level of TC and TG was significantly (*p* < 0.001) elevated, and HDL-C was significantly (*p* < 0.001) declined in the diabetic control compared to the normal control (Table 6). Administration of the latex for two weeks significantly (*p* < 0.05 at 200 mg/kg and *p* < 0.01 at 400 and 600 mg/kg) reduced level of TC and TG and increased (*p* < 0.01) the level of HDL-C in diabetic mice. Total cholesterol and triglyceride reducing effect of the latex at 200 mg/kg dose was lower than 5 mg/kg glibenclamide (*p* < 0.001).

## 4. Discussion

Antidiabetic activity of the leaf latex of *Aloe pulcherrima* was evaluated *in vitro* and *in vivo* models that offer useful evidence for the development of novel plant-based antidiabetic drugs [2, 6, 24].

In this study, the leaf latex of *A. pulcherrima* showed concentration-dependent antioxidant activity in DPPH assay. This finding is concordat with free radical scavenging activities of the latex of *Aloe schelpei, Aloe megalacantha*, and *Aloe monticola* in DPPH assay [25, 34, 39]. Oxidative stress is a decisive factor in the pathogenesis of diabetes mellitus. Reactive oxygen species act as a second messenger that activates nuclear factor kappa B (NF-*κ*B), necrosis factor-*α* (TNF-*α*), and interleukins (ILs) which reduces the expression of GLUT 4 and activates intracellular adhesion molecule-1 to stimulate the growth of insulin resistance [40–43]. Therefore, preventing oxidative stress might be one approach to reduce onset of diabetes and its complications [24, 43, 44]. Antioxidants, such as *α*-lipoic acid, ubiquinone, and flavonoids prevent diabetic onset and ameliorate its complications [43, 45].

The leaf latex of *A. pulcherrima* showed sucrase, maltase, and *α*-amylase inhibitory effects. Enzyme inhibition capacity of the latex was concentration dependent, and greater inhibition was observed on sucrase (IC_50_ = 2.92 *μ*g/ml) relative to maltase (IC_50_ = 11.81 *μ*g/ml) and *α*-amylase (IC_50_ = 14.92 *μ*g/ml) enzymes. This result is in line with concentration dependent *α*-amylase inhibitory effect of the leaf latex and isolated compounds of *Aloe megalacantha* and *Aloe monticola* [25]. The successful prevention or control of postprandial hyperglycemia is through inhibition of *α*-glucosidase and *α*-amylase, [26] since these enzymes facilitate carbohydrate digestion and absorption. *α*-Glucosidase is the most important enzyme for digestion and absorption of starch and sucrose [2, 24]. Modern *α*-glucosidase and *α*-amylase inhibitors have limited efficacy and induces deleterious effects [24, 25]. Therefore, plant-derived bioactive molecules may serve as novel alternatives in postprandial glycemic control.

Enzyme inhibitory effect of the latex calls for further investigation in normoglycemic, oral glucose-loaded, and streptozotocin-induced diabetic mice. Measuring blood glucose level was the most reliable parameter in all models. Body weight and serum lipid level were evaluated in the streptozotocin-induced diabetic model [24, 36].

The low dose of (200 mg/kg) the latex significantly (*p* < 0.05) induced hypoglycemia after 4 h, but did not sustain the effect for 6 h possibly due to early elimination from body. The medium and high doses of the latex brought significant hypoglycemia after 4 h and 6 h in the normoglycemic model. Hypoglycemic effect of glibenclamide was significant after 2, 4, and 6 h. This indicated that the leaf latex and glibenclamide changed blood glucose level though the same mechanism of action (increased insulin release or insulin-like effect). This finding is concordant with significant hypoglycemic effect of the leaf gel of *Aloe vera* and the latex of *A. megalacantha* in the normoglycemic model [34, 46]. Aloe species contain poly and monosaccharides that play blood glucose reduction by increasing insulin level, and hence, the plants show hypoglycemic activities [47, 48].

In this study, medium and high doses of the latex significantly (*p* < 0.001) reduced blood glucose level after one and two hours of oral glucose loading in the glucose tolerance test. Glucose reducing effect of the low dose was delayed and significant (*p* < 0.01) after two hours. The result showed that medium and high doses of the latex had fast onset and greater glucose reduction. This is comparable with significant postprandial glucose reducing effect of the leaf latex of *Aloe megalacantha* [34].

Administration of the latex for two weeks to STZ-induced diabetic mice showed prominent blood glucose reducing compared to diabetic control. Glucose reduction effect was increased with increasing the doses of the leaf latex of *A. pulcherrima* (*p* < 0.05, 200 mg/kg; *p* < 0.01, 400 mg/kg; and *p* < 0.001, 600 mg/kg on week one), indicating that low and medium doses of the latex had less cummulative effect on week one. Thus, cummulative glucose reduction with the latex was time and dose dependent (*p* < 0.01, low dose, and *p* < 0.001, medium and high dose on week two). The relative variation in blood glucose reduction among doses might be due to variation of the amount of secondary metabolite content in the leaf latex of *Aloe pulcherrima*. The findings showed that the plant was endowed with glucose lowering potential, and the effect was concordant with dose-dependent antidiabetic activity of the leaf latex of *Aloe megalacantha* [34]. In another study, administration of *Aloe vera* leaf gel at the doses of 200 and 400 mg/kg has been safe and effective as 50 mg/kg metformin [13]. Currently available antidiabetic agents contraindicated in renal and hepatic failure diabetic patients [14, 16]. *Aloe vera* leaf gel showed protective effect on diabetic-induced renal and liver damage, and hence, leaf gel of the plant and its derivative might be preferable in comorbid diabetic patients [13, 49].

In this study, significant BW increment was measured in diabetic mice treated with all doses of the leaf latex of *A. pulcherrima* and glibenclamide in STZ-induced diabetic mice. Compared to diabetic control, BW of the mice treated with all doses of the latex remarkably increased on week one and two. The study result indicated that the latex was endowed with preventive effect against diabetic-induced body weight loss.

Induction of diabetes by streptozotocin significantly elevated total cholesterol (TC) and triglycerides (TG) while decreasing high-density lipoprotein (HDL-C) compared to normal control. After two weeks of intervention with the leaf latex of *A. pulcherrima,* the lipid level of diabetic mice was significantly improved compared to diabetic control. All doses of the latex significantly reduced the levels of TC and TG, and increased the level of HDL-C. Thus, the latex of *Aloe pulcherrima* might serve as the potential candidate in the treatment of hyperglycemia induced dyslipidemia. The current result is in line with antidyslipidemic activity of the leaf of *Aloe vera* and *Aloe megalacantha* in STZ diabetic rodents [34, 48].

The finding showed that antidiabetic activity of the leaf latex of *Aloe pulcherrima* was statistically significant. Medium and higher doses of the latex showed comparable antidiabetic and antidyslipidemic activities with glibenclamide. Therefore, *Aloe pulcherrima* might serve as potential source for the development of the novel plant-based antidiabetic agents.

Flavonoids, glycosides, alkaloids, terpenoids, and others were present in the leaf latex of *Aloe pulcherrima* and have shown antidiabetic and antidyslipidemic activity in various plant extracts [9, 11, 13, 24, 30, 34, 50]. Many bioactive compounds isolated from *Aloe* species have sugar moiety and structurally resemble with glucose which is a key factor for insulin release [51]. Therefore, hypoglycemic, antihyperglycemic, and antidyslipidemic activities of the leaf latex of *Aloe pulcherrima* is possible due to a single or combined action of phytochemicals.

## 5. Conclusion

The result showed that the leaf latex of *Aloe pulcherrima* was endowed with a prominent blood glucose reducing effect. This finding offers a clue for the development of safe and effective herbal formulation to treat diabetes mellitus. Consequently, further study is required for characterization and isolation of bioactive compounds that have antidiabetic effect.

## Figures and Tables

**Table 1:** Phytochemical analysis result.

Phytochemical constituents	Result
Anthraquinones	+
Flavonoids	+
Saponins	+
Glycosides	+
Tannins	+
Phenols	+
Alkaloids	+
Steroids	−

Abbreviation: +, present; −, absent.

**Table 2:** The effect of the leaf latex of *Aloe pulcherrima* on glucose level in normoglycemic mice.

Groups	Blood glucose level (mg/dl)				
	0	1 h	2 h	4 h	6 h
10 ml/kg vehicle	90.26 ± 0.31	86.29 ± 0.75	78.72 ± 1.18	74.82 ± 1.71	74.46 ± 1.03
5 mg/kg·GB	90.25 ± 0.25	79.62 ± 0.11	64.89 ± 0.16 ^a2^	61.09 ± 0.29^a2^	55.75 ± 2.09 ^a3^
200 mg/kg·LL	89.33 ± 0.95	82.61 ± 1.42	76.78 ± 1.34	68.03 ± 1.33 ^a1^	72.49 ± 1.49
400 mg/kg·LL	89.43 ± 0.15	81.49 ± 0.96	76.19 ± 1.41	67.57 ± 2.17 ^a1^	62.65 ± 1.03 ^a2^
600 mg/kg·LL	91.11 ± 0.34	80.61 ± 0.46	74.22 ± 1.09	67.12 ± 0.85 ^a1^	61.11 ± 0.69 ^a2^

The result is expressed as mean ± standard error of the mean, *n* = 6; ^a^compared to vehicle; ^1^*p* < 0.05; ^2^*p* < 0.01; ^3^*p* < 0.001; GB, glibenclamide; LL, leaf latex.

**Table 3:** Effect of the leaf latex of *A. pulcherrima* on the glucose tolerance test in mice.

Groups	Blood glucose level (mg/dl)			
	0 h	0.5 h	1 h	2 h
10 ml/kg vehicle	89.13 ± 0.31	156.95 ± 0.67	142.85 ± 0.55	125.51 ± 0.81
5 mg/kg glibenclamide	88.37 ± 1.19	157.23 ± 0.71	114.68 ± 1.33^a3^	91.47 ± 0.27^a3^
200 mg/kg·LL	89.33 ± 0.45	158.24 ± 1.31	129.57 ± 0.61	107.50 ± 1.09^a2^
400 mg/kg·LL	87.67 ± 0.26	159.12 ± 0.29	120.28 ± 0.72^a3^	98.61 ± 0.37^a3^
600 mg/kg·LL	90.21 ± 1.22	157.87 ± 0.51	118.25 ± 0.27^a3^	94.72 ± 0.51^a3^

The result is presented as mean ± standard error of the mean, *n* = 6; ^a^compared to vehicle; ^1^*p* < 0.01; ^2^*p* < 0.001; LL, leaf latex.

**Table 4:** The effect of the leaf latex of *Aloe pulcherrima* on glucose level in diabetic mice.

Groups	Blood glucose level (mg/dl)		
	Pretreatment	Week one	Week two
10 ml/kg vehicle NC	89.17 ± 1.56	88.86 ± 1.89	90.56 ± 1.35
10 ml/kg vehicle DC	258.47 ± 1.39	262.62 ± 1.09	263.75 ± 4.23
5 mg/kg glibenclamide	260.56 ± 3.37	240.17 ± 2.50 ^a3b1^	233.33 ± 1.89 ^a3b1^
200 mg/kg·LL	259.33 ± 2.43	248.47 ± 2.44 ^a1c1^	241.27 ± 1.96^a2c1^
400 mg/kg·LL	261.49 ± 2.98	247.28 ± 2.99 ^a2^	237.15 ± 4.09^a3^
600 mg/kg·LL	259.89 ± 2.50	245.45 ± 2.50^a3b1^	234.21 ± 0.72^a3^

The result is expressed as mean ± standard error of the mean, *n* = 6; ^a^compared to diabetic control; ^b^to 200 mg/kg; ^c^to 5 mg/kg glibenclamide; ^1^*p* < 0.05; ^2^*p* < 0.01; ^3^*p* < 0.001. DC, diabetic control; LL, leaf latex; NC, normal control.

**Table 5:** The effect of the latex of *Aloe pulcherrima* on body weight of diabetic mice.

Groups	BW (gram)		
	Pretreatment	Week one	Week two
10 ml/kg vehicle NC	27.82 ± 0.32	28.64 ± 0.38	28.94 ± 0.38
10 ml/kg vehicle DC	24.59 ± 0.59	23.08 ± 0.74	22.94 ± 0.45
5 mg/kg glibenclamide	24.23 ± 0.53	28.76 ± 0.56^a3^	28.87 ± 0.36 ^a3^
200 mg/kg·LL	23.19 ± 0.65	26.65 ± 0.49 ^a1^	26.15 ± 0.43 ^a1^
400 mg/kg·LL	23.47 ± 0.63	26.91 ± 0.60 ^a1^	27.23 ± 0.61 ^a1^
600 mg/kg·LL	25.07 ± 0.33	27.72 ± 0.34 ^a1^	27.82 ± 0.35 ^a1^

The result is expressed as mean ± standard error of the mean, *n* = 6; ^a^compared to diabetic control; ^1^*p* < 0.01; ^2^*p* < 0.001. BW, body weight; DC, diabetic control; LL, leaf latex; NC, normal control.

**Table 6:** The effect of the leaf latex of *Aloe pulcherrima* on serum lipid level in diabetic mice.

Groups	Serum lipid level		
	TC	TG	HDL-C
10 ml/kg vehicle NC	88.50 ± 5.86	96.83 ± 5.86	38.83 ± 1.49
10 ml/kg vehicle DC	187.17 ± 2.44	175.83 ± 2.97	26.67 ± 2.02
5 mg/kg glibenclamide	94.50 ± 2.09^a3b1^	105.76 ± 7.80^a3b1^	37.67 ± 0.71^a3^
200 mg/kgvLL	179.00 ± 1.71^a1c1^	154.33 ± 2.65^a1c1^	34.17 ± 1.08^b2^
400 mg/kg·LL	178.33 ± 1.58^a2^	154.17 ± 1.51^a2^	34.33 ± 1.15^b2^
600 mg/kg·LL	178.30 ± 1.02^a2^	153.33 ± 4.34^a2^	36.67 ± 0.71^b2^

Values are expressed as mean ± standard error of the mean, *n* = 6; ^a^compared to diabetic control; ^b^to 200 mg/kg; ^c^to 5  mg/kg. ^1^*p* < 0.05; ^2^*p* < 0.01; ^3^*p* < 0.001. DC, diabetic control; HDL-C, high-density lipocholestrol; NC, normal control; LL, leaf latex; TC, total cholesterol; TG, triglyceride.

## Data Availability

All the datasets used or analyzed during the present study are available from the corresponding author on reasonable request.

## Abbreviations

EPHI: Ethiopian Public Health Institute
GLUT 4: Glucose transporter 4
TC: Total cholesterol
TG: Triglycerides.
